# Gluten cross contact in oats: retrospective database analysis 2011 to 2023

**DOI:** 10.3389/fnut.2023.1284636

**Published:** 2023-11-22

**Authors:** Tricia Thompson, Amy Keller

**Affiliations:** ^1^Gluten Free Watchdog, LLC, Manchester, MA, United States; ^2^Mary Rutan Hospital, Bellefontaine, OH, United States

**Keywords:** celiac disease, gluten-related disorders, gluten cross-contact, oats, gluten

## Abstract

It is long-established that oats are at substantial risk for cross contact with gluten-containing grain. Specially processed gluten-free oats, whether purity protocol or mechanically/optically sorted, made it possible for this grain to be included in a gluten-free diet in the U.S. Gluten Free Watchdog (GFWD) (Manchester, Massachusetts, United States) has been assessing the gluten content of labeled gluten-free foods since 2011. In 2022, there was an apparent increase in the number of oat products testing with quantifiable gluten at or above 5 mg/kg or parts per million (ppm). The purpose of the present study was to assess the levels of gluten in foods containing oats to determine if there were any trends. In this retrospective database analysis, GFWD product test reports from April 2011 to May 1, 2023 were searched using the term “oat.” The search identified 213 individual packages of food that contained the word “oat” in the ingredients list. The test results for these packages of food were reviewed. Of these, 24 (11%) tested with quantifiable gluten greater than or equal to 5 mg/kg (ppm). The percentage of oats testing with quantifiable gluten varied per year but spiked in 2022 at 35%. It is not possible to know for certain what caused this increase. The drought during the oat growing season of 2021 could be a major factor. This drought impacted oat crops in both the US and Canada and led to one of the worst oat crops going back over 150 years. One limitation of this study is that it was a retrospective analysis. Different numbers of oat products were tested each year and these were often different brands of oats and different oat formulations. To assess the level of gluten cross contact in oats going forward a much larger prospective study should be conducted.

## Introduction

Under the U.S. Food and Drug Administration’s (FDA) gluten-free labeling rule (1) oats are allowed in foods labeled gluten-free. There is no requirement for oats to be specially processed. However, like all foods carrying a gluten-free claim, single ingredient oat products and products containing oats must have a level of gluten below 20 mg/kg or 20 parts per million (ppm). The FDA’s gluten-free rule applies to gluten found naturally in a food as well as gluten cross contact (e.g., as might be found in oats).

It is long-established that oats are at substantial risk for cross contact with gluten-containing grain (2). At the farm level, this risk may be due to crop rotation with wheat, barley, or rye, proximity to fields growing gluten-containing grain, and/or shared harvesting equipment, among other things (3).

To help decrease the risk of gluten cross contact in oats, farmers in North America came together in 2003 to develop what was termed a gluten-free purity protocol for oats (4). While the exact protocol may vary among farms, steps are in place to control everything from the seed planted to the equipment used for harvesting to the plant used for processing (5). In addition to oats grown under a gluten-free purity protocol, oats for the gluten-free market may be mechanically and optically sorted to remove errant wheat, barley, and rye at the processing plant (6). Specially processed gluten-free oats, whether purity protocol or sorted, made it possible for this grain to be included in a gluten-free diet in the U.S.

Many people with celiac disease enjoy eating gluten-free oats, and the number of labeled gluten-free foods containing oats has increased substantially in recent years (7). Incorporating oats into the diet can help people with celiac disease increase their whole grain consumption and meet nutritional goals for micronutrients and fiber. In particular, increased beta-glucan intake, the soluble fiber found in oats, may reduce the risk of heart disease (8).

Gluten Free Watchdog (GFWD) (Manchester, Massachusetts, United States) has been assessing the gluten content of labeled gluten-free foods since 2011. We have previously reported that the vast majority of foods testing at or above 20 mg/kg (ppm) as tested through GFWD are grain-based foods. Oat products account for almost half of these products (9). Regardless, the overall percentage of foods testing at or above 20 mg/kg (ppm) is low—approximately 4% at last check.

While oat-containing products make up a substantial portion of foods testing out of compliance with the gluten-free labeling rule, gluten-containing grain may escape detection when oats are tested for gluten. This is due to the heterogeneous nature of gluten cross-contact within oats (10–12). Any gluten containing grain present in the test sample may not be uniformly distributed.

The purpose of the present retrospective database analysis was to assess the levels of gluten in foods containing oats to determine if there were any trends. This assessment was triggered in part by an apparent increase in 2022 in the number of oat products, including purity protocol, testing with quantifiable gluten as assessed through GFWD. One of the four purity protocol oat suppliers included in a carefully vetted gluten-free purity protocol listing maintained by GFWD (5) had one of their branded oat products test between 6 and 32 mg/kg (ppm) of gluten. Another supplier had one of their branded oat products test between 9 and 15 mg/kg (ppm) of gluten. While the latter product tested within the allowed level of gluten under the FDA’s gluten-free labeling rule, these combined findings raised levels of concern about oats. Additionally, we were advised by a third supplier, that because of the 2021 drought that affected North America, the purity protocol standards they provided to Gluten Free Watchdog (and that were published online) were not 100% adhered to, although followed as closely as possible (5).

## Methods

In this retrospective database analysis, GFWD product test reports from April 2011 to May 1, 2023 were searched using the search term “oat.” The search identified 213 individual packages of food that contained the word “oat” in the ingredients list. The test results for these packages of food were reviewed.

Products tested through GFWD are based almost exclusively on requests from subscribers. Test requests are made for any number of reasons, including peace of mind regarding a favorite product or concern that a food may be “contaminated” with gluten.

Products were sent to the ISO/IEC 17025 accredited lab, Bia Diagnostics, LLC in Colchester, VT, United States. Oat-containing foods were tested using the Ridascreen Gliadin sandwich R5 enzyme-linked immunosorbent assay (ELISA) Mendez method (Ridascreen Gliadin R7001) and extracted with the cocktail solution (Art. No. R7006—official Mendez method) following the kit manufacturer instructions (R-Biopharm, Darmstadt, Germany) (10). This is one of two assays FDA states they will use when products are tested as part of gluten-free rule enforcement (13). At least two test portions were tested from each homogenized food sample.

Kit manufacturer instructions for testing oats for gluten using the sandwich R5 ELISA currently state to homogenize at least a 200-gram food sample (10). At least 1-gram food portions from this homogenized sample should be tested. Typically (and including for oats up until approximately 2016) foods are tested by homogenizing a 50-gram food sample and then testing food portions weighing 0.25 grams. According to R-Biopharm, increased test portions are recommended for oats because any gluten-containing grain present in a sample will not be evenly distributed, and it is difficult to homogenize oat samples that may contain these grains. Homogenizing a larger sample and testing larger test portions increases the likelihood that the presence of any gluten-containing grain will be detected.

The lower limit of quantification for the Ridascreen Gliadin R5 ELISA is 5 mg/kg (ppm) of gluten. In this analysis, only food packages that tested at or above 5 mg/kg (ppm) (on either initial testing and/or after being reground/re-homogenized or resampled) are included.

## Results

**Figure 1:**
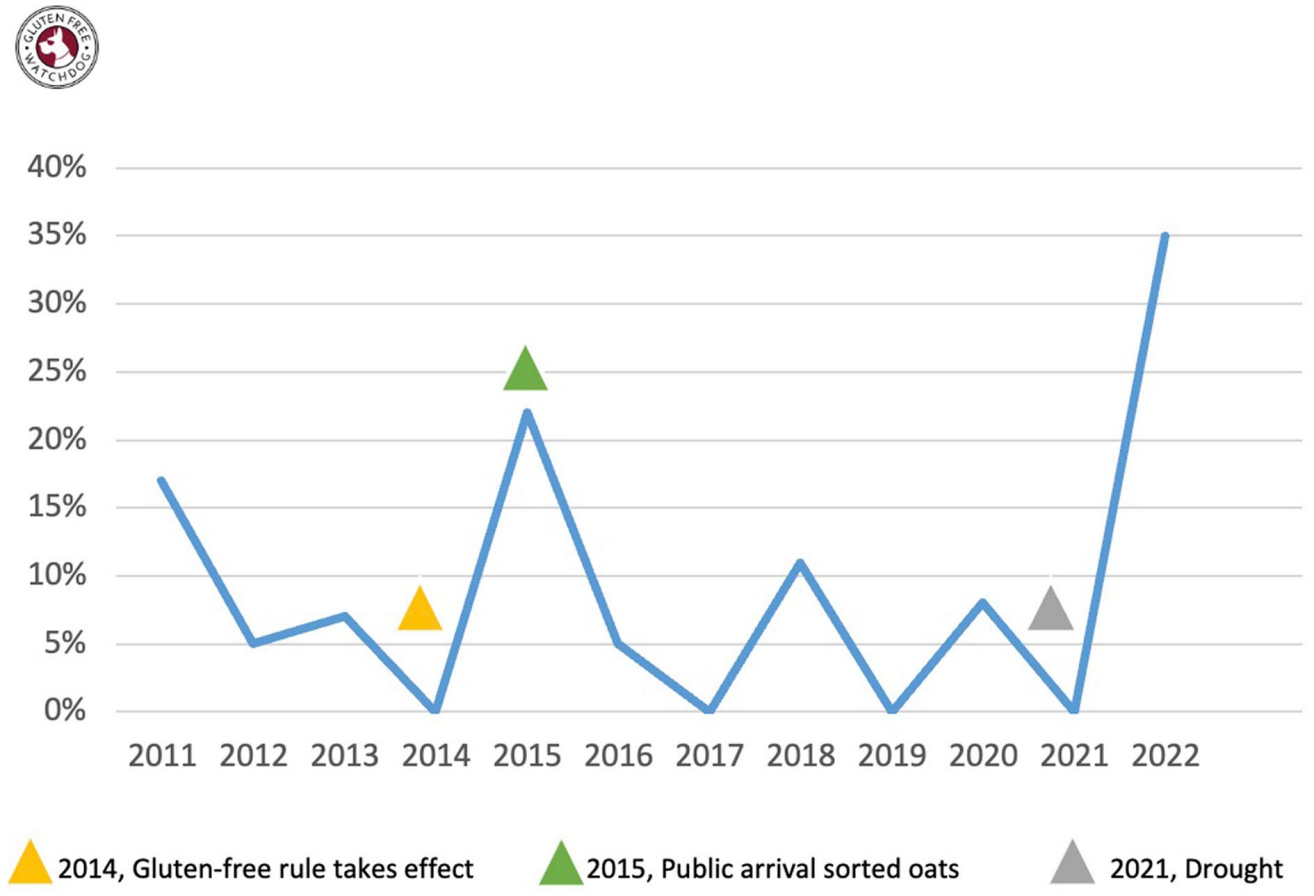
Percentage of oats testing with quantifiable gluten April 2011 through December 2022.

213 products (i.e., individual packages) contained the word oat in the ingredients list. Of these, 24 (11%) tested with quantifiable gluten greater than or equal to 5 mg/kg (ppm). The percentage of oats testing with quantifiable gluten varied per year from a low of zero in years 2014, 2017, 2019, and 2021 to a high of 35% in 2022 (Table 1; Figure 1). The year the gluten-free labeling rule took effect (2014), 0/13 packages tested with quantifiable gluten (Figure 1). This was followed by a spike in 2015 with 22% of packages testing with quantifiable gluten. Of note, during this time there was a more widespread use of mechanically and optically sorted oats (6). Levels then held fairly steady between zero and 11% until another spike in 2022 when 35% tested with quantifiable gluten. Of note, the drought of 2021 impacted areas where oats are grown (14).

**Table 1:** Products testing with quantifiable gluten by year (*n* = 24).

Year/summary	Product	Mg/Kg (ppm) gluten range	Oat source	Certified GF?
2011/(3/18 products);	Hot Cereal (oats first ingredient)	28–30	Unknown	Yes
	Hot Cereal (oats first ingredient)	> 100	Unknown	Yes
	Hot Cereal (oats first ingredient)	18–26	Unknown	Yes
2012 (1/21)	Hot Cereal (oats first ingredient)	63–73	Unknown	Yes
2013 (1/14)	Crumb Mixture (oats first ingredient)^a^	9 – > 80 (9, 16, 22, > 80)	Standard	No
2015 (5/23)	Granola (oats first ingredient)^b^	6–30	Unknown	No
	Rolled Oats (oats only ingredient)	72 – > 80	Unknown	No
	Cold Cereal (oats first ingredient)	6–7	Sorted	No
	Bar (oats as one of many ingredients)	12–15	Unknown	No
	Granola (oats second ingredient)^c^	16–26	Standard	No
2016 (1/21)	Oat matzoh (oats first ingredient)	12–13	Purity protocol	No
2018 (1/9)	Cookie (oats first ingredient)	10–15	Certified GF	No
2020 (1/13)	Bread mix (oats first ingredient)^d^	6–44	Sorted	No
2022 (9/26)	Oat flour (oats only ingredient)^e^ (sample)^f^	7–22	Purity protocol	Yes
	Oat flour (oats only ingredient)	6–14	Purity protocol	Yes
	Oat flour (oats only ingredient)	12–16	Purity protocol	Yes
	Oat flour (oats only ingredient)	9–32	Purity protocol	Yes
	Rolled Oats (oats only ingredient) (sample)^f^	9 – > 80 (9, 15, > 80)	Purity protocol	Yes
	Rolled Oats (oats only ingredient)	6 – > 80 (6, > 80, > 80)	Unknown	No
	Bread (oats first ingredient)	8–10	Unknown	No
	Bread (oats first ingredient)	6–13	Unknown	No
	Granola (oats first ingredient)^g^	71 – > 80	Unknown	Yes
2023 (2/8)	Oat flour (oats only ingredient) (sample)^f^	9–15	Purity Protocol	Yes
Through May 1	Oat flour (oats only ingredient) (sample)^f^	7–14	Unknown	No

a Oat source changed after testing.

b Label changed from “gluten-free” to “using gluten-free ingredients” after testing.

c Oat source changed after testing.

d Product recalled after testing.

e Safety alert issued by certifying agency for product represented in first four rows for 2022.

f Consumer sample sent to Gluten Free Watchdog for testing after testing positive for gluten using an LFD.

g Safety alert issued by certifying agency.

Of the 24 packages that tested with quantifiable gluten, oats were either the only ingredient or the first ingredient in all but 2 products. Seven packages of oat products were purity protocol (4 different products/3 brands), 2 packages were mechanically/optically sorted oats, 2 packages were standard oats, and 1 package used certified oats even though the product was not certified gluten-free. The source of the remaining oats was unknown at the time of testing.

In this analysis, products containing purity protocol oats testing with quantifiable gluten is a relatively recent occurrence. Six of seven packages of purity protocol oat products (3 different products, 2 brands) testing with quantifiable gluten occurred in 2022/23; 1 occurred in 2016. Products using purity protocol oats also tended to be certified gluten-free in this analysis. While purity protocol oat products testing with quantifiable gluten in 2022/23 came from 2 brands, these particular manufacturers are also suppliers of purity protocol oats. In other words, they sell purity protocol oats to many other manufacturers.

While overall, 11% of packages of oat products tested with quantifiable gluten, only 7% had test portions that tested at/above 20 mg/kg (ppm). Some results were widely divergent with at least 1 test portion testing >80 mg/kg (ppm). This can occur as a result of the heterogeneous nature of gluten cross contact and difficulty homogenizing some samples. In these instances, the test portion results have been provided in addition to a range.

## Discussion

### Potential causes of increased levels of gluten in oats

It is not possible to know for certain what caused the 2022 increase in oat products, including purity protocol oats, testing at levels of gluten at/above 5 mg/kg (ppm). The drought during the oat growing season of 2021 could be a major factor. This drought impacted oat crops in both the US and Canada and led to one of the worst oat crops going back over 150 years (15). According to a June, 2022 article in *Food Safety Magazine*, the output of oat crops was reduced by half as a result of the drought (16). Also in 2022, the Gluten Intolerance Group posted about the shortage of gluten-free oats, warning consumers about the risk of unsafe levels of gluten in oats related to these shortages. They wrote that the issues were impacting both purity protocol and mechanically sorted oats (17). In addition, supply chain disruptions due to the Covid pandemic may have further exacerbated issues caused by the drought. Market reports during the pandemic suggested that among other things, consumers were eating more oatmeal (18).

It is possible that the drought contributed to an increase in infesting weeds that may contain gluten, such as ryegrass (not to be confused with the young grass of the rye plant) (19). While some evidence suggests that gluten-like peptides are present in ryegrass, it is unclear whether this gluten, even if present in oats tested in 2022, would have been detected during testing using the R5 ELISA (19). This is an area that requires further exploration.

It is not known how long GFWD will continue to see increased levels of oat products testing with quantifiable gluten. Oats that were packaged in 2022 from the 2021 growing season will remain on store shelves for a period of time and in the pantries of consumers for longer still. There is also a market report suggesting there has been a fall in oat prices and the potential for fewer oats to be grown (20). According to a July 2023 pre-harvest crop report from Avena Foods, lower yields are expected for oats, and they will be thin and have poor milling quality (21). However, preliminary data for oats tested after completion of the current analysis, suggests that levels of gluten cross contact in products tested through Gluten Free Watchdog may be decreasing.

### Recommendations

There is an increasing need for oats as the number of labeled gluten-free foods containing oats continues to grow (7). It is very important for all oat suppliers to put steps in place to protect gluten-free consumers even when droughts, supply chain issues, etc. impact the availability of gluten-free oats. During such times, oat suppliers should communicate any changes to their oat protocols (e.g., if the oats are sold as purity protocol but the protocol was altered after the fact to allow demand to be met, manufacturers should be notified). A change in protocol could very well impact the level of gluten in a product, and manufacturers should receive immediate notification of these changes so additional gluten testing of the final product can be conducted.

At all times, manufacturers should be mindful of the issues around testing oats for gluten. Testing can be problematic due to what has been described by researches as kernel-based gluten contamination (11, 12). For example, the percentage of labeled gluten-free oats with quantifiable gluten at or above 20 ppm, may be underestimated, depending upon sampling. In one of their studies, Fritz et al. (11) conducted additional testing on samples of oats initially testing between 5 ppm and less than 20 ppm gluten based on a single test portion. Additional testing found that at least 9 of these 20 samples contained on average above 20 ppm of gluten based on testing multiple extractions from each sample. This despite the fact that the first extraction tested contained less than 20 ppm of gluten.

Practitioners working with clients with celiac disease should remain aware of the current issues with gluten-free oats. Single-ingredient oat products may require the greatest vigilance. Generally speaking, multi-ingredient products containing oats tend to contain lower levels of gluten when tested. This is because any gluten present tends to be “diluted” because of other ingredients. Some practitioners may choose to recommend only those oats grown under a gluten-free purity protocol. However, practitioners should be aware that in times of drought and supply chain issues, a purity protocol is only as good as the extent to which it is followed.

Patients with celiac disease will likely be asking questions now and in the future about whether oats should remain a part of their gluten-free diet. Uncertainty regarding how best to answer this question will likely remain for practitioners. Overall, 7% of test portions in the current retrospective database review tested at gluten levels of 20 ppm or above. Whether this is problematic may vary depending upon the eating patterns of individuals. For example, if a consumer with celiac disease consistently eats a product with gluten levels above 80 mg/kg (ppm), for each 28.3-gram amount (1 ounce) consumed, they may be ingesting at least 2.28 mg of gluten. For grain foods such as oatmeal and granola, it is very easy to ingest a few ounces. In the US, 10 mg of gluten per day is generally considered by experts to be a tolerable amount for most individuals with celiac disease (22).

### Study limitations

One benefit of the current assessment is that it looked at oats tested by the same group—GFWD, and at the same lab—Bia Diagnostics over the past 12 years. One limitation is that it was a retrospective analysis. Different numbers of oat products were tested each year and these were often different brands of oats and different oat formulations. As a result, no statements can be made regarding the extent of gluten cross-contact within any brand or variety of oats. Another limitation is that the protocol recommended by R-Biopharm for testing oats with the sandwich R5 ELISA changed over the 12-year test period of this assessment. In 2016 GFWD started homogenizing larger food samples and testing larger test portions. Also, because this is a retrospective analysis of products requested for testing by subscribers to GFWD, products tested may not be representative of oat products in the US market. The number of products tested represents a very small portion of all oat products available. To assess the level of gluten cross contact in oats going forward a much larger prospective study should be conducted.

## Conclusion

At Gluten Free Watchdog there was a spike in oat products testing with quantifiable gluten in 2022 that carried over into early 2023. It is unclear what led to this spike but a major factor may have been the drought that occurred in 2021. It is hoped that going forward, the percentage of oats with quantifiable gluten will fall at least to the mean level of 7% seen pre-drought. It is also hoped that when there is disruption to the oat supply, any changes by suppliers to their gluten-free oat protocols will be communicated not only to manufacturers and certifying organizations, but ultimately to consumers.

## Data availability statement

The data analyzed in this study is subject to the following licenses/restrictions: Gluten Free Watchdog has been testing food for gluten since 2011. Data is behind a paywall. Pertinent data on oats is included in this manuscript. Requests to access the Gluten Free Watchdog database should be directed to Tricia Thompson; this database cannot be accessed without a subscription to Gluten Free Watchdog.

## Author contributions

TT: Conceptualization, Data curation, Investigation, Methodology, Writing – original draft. AK: Conceptualization, Data curation, Investigation, Methodology, Writing – review & editing.

## References

[1] U.S. Food and Drug Administration. Federal Register. Food labeling; gluten-free labeling of Foods (2013). Available at: https://www.federalregister.gov/documents/2013/08/05/2013-18813/food-labeling-gluten-free-labeling-of-foods (Accessed June 6, 2023). 23923139

[2] Thompson T. Gluten contamination of commercial oat products in the United States. N Engl J Med. (2004) 351:2021–2. doi: 10.1056/NEJM200411043511924, PMID: 15525734

[3] Thompson T. Oats and the gluten free diet. J Am Diet Assoc. (2003) 103:376–9. doi: 10.1053/jada.2003.50044 12616264

[4] Montana Gluten Free. Our oats. Gluten free purity protocol. Available at: https://www.montanaglutenfree.com/shop/montana-gluten-free-oat-purity-protocol (Accessed June 6, 2023).

[5] Thompson T. Gluten Free Watchdog. Oats produced under a gluten-free purity protocol: Listing of suppliers and manufacturers. Available at: https://www.glutenfreewatchdog.org/news/oats-produced-under-a-gluten-free-purity-protocol-listing-of-suppliers-and-manufacturers (Accessed August 3, 2023).

[6] General Mills. Simply gluten-free. Available at: https://www.cheerios.com/gluten-free (Accessed June 6, 2023).

[7] Future Market Insights. Gluten-free oats market outlook (2023 to 2033) (2023). Available at: https://www.futuremarketinsights.com/reports/gluten-free-oats-market (Accessed August 3, 2023).

[8] The Food and Drug Administration. Code of Federal Regulations 21CFR101.81. Health claims: soluble fiber from certain foods and risk of coronary heart disease (CHD) (1997). Available at: https://www.accessdata.fda.gov/scripts/cdrh/cfdocs/cfcfr/cfrsearch.cfm?fr=101.81 (Accessed August 3, 2023).

[9] Thompson T. What we’ve learned from 15 years of testing food for gluten & 8 years running gluten free watchdog (2020). Available at: https://www.glutenfreewatchdog.org/news/what-weve-learned-from-15-years-of-testing-food-for-gluten-8-years-running-gluten-free-watchdog/ (Accessed August 3, 2023).

[10] R-Biopharm AG. RIDASCREEN® gliadin art. no. R7001. Instructions (2021). Available at: https://food.r-biopharm.com/wp-content/uploads/r7001-gliadin-2021-10-11.pdf (Accessed June 6, 2023).

[11] Fritz RD Chen Y Contreras V. Gluten-containing grains skew gluten assessment in oats due to sample grind non-homogeneity. Food Chem. (2017) 216:170–5. doi: 10.1016/j.foodchem.2016.08.031 27596406

[12] Fritz RD Chen Y. Kernel-based gluten contamination of gluten-free oatmeal complicates gluten assessment as it causes binary-like test outcomes. Food Sci Technol. (2017) 52:359–65. doi: 10.1111/ijfs.13288

[13] U.S. Food and Drug Administration. Questions and answers on the gluten-free food labeling final rule (2022). Available at: https://www.fda.gov/food/food-labeling-nutrition/questions-and-answers-gluten-free-food-labeling-final-rule (Accessed June 6, 2023).

[14] Gro Intelligence. Cereal and oat Milk makers feel the crunch from oats shortage (2021). Available at: https://www.gro-intelligence.com/insights/cereal-and-oat-milk-makers-feel-the-crunch-from-oats-shortage (Accessed June 6, 2023).

[15] Hirtzer M Carey D. Bloomberg. Drought pushes U.S. oat crop to lowest in records Back to 1866 (2021). Available at: https://www.bloomberg.com/news/articles/2021-07-12/drought-pushes-u-s-oat-crop-to-lowest-in-records-back-to-1866#xj4y7vzkg (Accessed June 6, 2023).

[16] Allred L. Food safety magazine. Best Practices for Sourcing Gluten-free Oats when Supply is Limited (2022). Available at: https://www.food-safety.com/articles/7820-best-practices-for-sourcing-gluten-free-oats-when-supply-is-limited (Accessed May 30, 2023).

[17] Gluten Intolerance Group. The shortage of gluten free oats (2022). Available at: https://gluten.org/2022/01/31/the-shortage-of-gluten-free-oats/ (Accessed October 20, 2023).

[18] Smith B. Business Insider. Americans are eating more cereal and oatmeal during the coronavirus pandemic (2020). Available at: https://www.businessinsider.in/retail/news/americans-are-eating-more-cereal-and-oatmeal-during-the-coronavirus-pandemic/articleshow/77207073.cms (Accessed October 24, 2023).

[19] Escobar-Correas S Broadbent J Andraszek A Stockwell S Howitt C Juhász A. Perennial ryegrass contains gluten-like proteins that could contaminate cereal crops. Front Nutr. (2021) 8:708122. doi: 10.3389/fnut.2021.708122, PMID: 34395501 PMC8355629

[20] Peleshaty A. The Western producer. Uncertain year ahead for oats as price collapses (2023). Available at: https://www.producer.com/news/uncertain-year-ahead-for-oats-as-price-collapses (Accessed June 6, 2023).

[21] Avena Foods. Mike’s pre-harvest crop report 2023 (2023). Available at: https://mailchi.mp/avenafoods/avena-pre-seeding-report-spring-6061999?e=018b995a1f (Accessed August 3, 2023).

[22] Catassi C Fabiani E Iacono G D'Agate C Francavilla R Biagi F. A prospective, double-blind, placebo-controlled trial to establish a safe gluten threshold for patients with celiac disease. Am J Clin Nutr. (2007) 85:160–6. doi: 10.1093/ajcn/85.1.160, PMID: 17209192

